# Multi-decadal to centennial hydro-climate variability and linkage to solar forcing in the Western Mediterranean during the last 1000 years

**DOI:** 10.1038/s41598-018-35498-x

**Published:** 2018-11-28

**Authors:** Yassine Ait Brahim, Jasper A. Wassenburg, Francisco W. Cruz, Abdelfettah Sifeddine, Denis Scholz, Lhoussaine Bouchaou, Emilie P. Dassié, Klaus P. Jochum, R. Lawrence Edwards, Hai Cheng

**Affiliations:** 1 Insistute for Global Environmental Change, Xi’an Jiaotong University, Xi’an, China; 2 Climate Geochemistry Department, Max Planck Institute for Chemistry, Mainz, Germany; 3 Instituto de de Geociências, Universidade de São Paulo, São Paulo, Brazil; 4 IRD-Sorbonne Universités (UPMC, CNRS, MNHN) UMR LOCEAN, Centre IRD, Bondy, France; 5 Institute of Geoscience, University of Mainz, Mainz, Germany; 6 Laboratory of Applied Geology and Geo-Environment, Ibn Zohr University, Agadir, Morocco; 7 EPOC, UMR 5805, CNRS, University of Bordeaux, Pessac, France; 8 Department of Earth Sciences, University of Minnesota, Minneapolis, MN 55455 USA

**Keywords:** Palaeoclimate, Geochemistry

## Abstract

Here we present a new composite record from two well-dated speleothem records from two caves in Northern Morocco. The high-resolution record covers the last millennium allowing to detect multi-decadal to centennial periodicities. Over the industrial period, δ^18^O values of our speleothems are shown to be dominated by the main mode of decadal variability in the North Atlantic region: the North Atlantic Oscillation (NAO). Statistical analyses confirm the previously reported multi-decadal variability related to the influence of the Atlantic Multidecadal Oscillation (AMO) in the region. High power and persistent centennial-scale periodicities, similar to the Vries-Suess 200-year solar cycle, are observed as well. Indeed, comparison between solar activity reconstructions and our record confirms the in-phase relationship on centennial time-scales. Low δ^18^O values, and hence negative phases of NAO that bring precipitation towards the Western Mediterranean, are observed during well-known solar minima periods. The results are consistent with previous models which describe low irradiance as a  trigger for southward shifts of precipitation-bearing westerlies during winter.

## Introduction

The Western Mediterranean, located at the north-eastern side of the Azores High and hence under the direct influence of the North Atlantic Oscillation (NAO)^1,2^, is considered as one of the most sensitive regions to drought^3^. Climate models anticipate more drought and reduced annual precipitation in this region in the future^4,5^.

At seasonal to interannual time scales, the NAO is indeed the main atmospheric phenomenon that modulates the strength and the direction of the westerly winds and consequently affects the climate patterns in the Western Mediterranean, with severe socio-economic and ecological consequences^6^. Previous studies have shown that the Western Mediterranean climate is highly influenced by the NAO, whereas positive and negative NAO phases result in droughts and higher than normal rainfall in the region^1,2,7^. Additionally, the Atlantic multidecadal oscillation (AMO) has been shown to impact climate in the North Atlantic region. Speleothem records in Morocco covering the last 1000 years have revealed the presence of multidecadal hydro-climate variability consistent with the AMO known periodicity^8^.

Information on climate variability in the Western Mediterranean remains however scarce due to the lack of long instrumental datasets. High-resolution paleoclimate reconstructions in this region are thus essential to increase our understanding of hydroclimate variability on various timescales and to discuss potential forcing mechanisms and related atmospheric circulation changes in the remote past beyond the industrial period.

**Figure 1:**
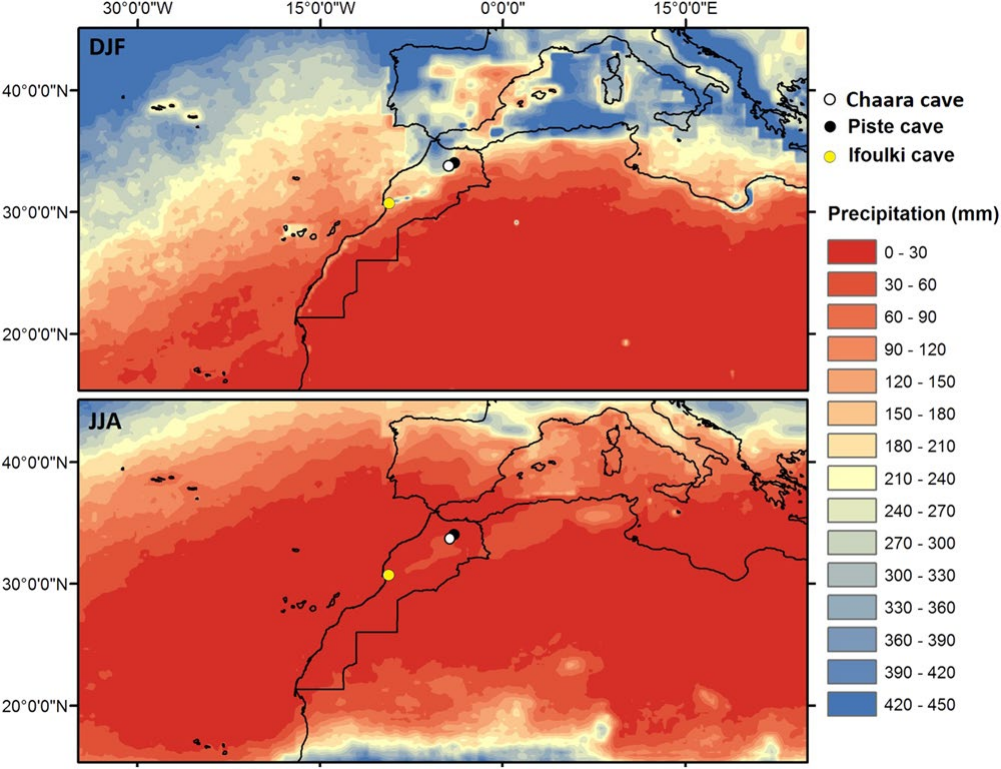
Rainfall amount in Northwest Africa and Southwestern Europe (mm/month) during the winter (December–January–February; DJF) and the summer (June–July–August; JJA) seasons, calculated from the “TRMM 3B42 daily V7” data^45^ for the period 1998–2015. Locations of paleoclimate records discussed in the paper are indicated: Chaara cave (white circle), Piste cave (black circle^13^) and Ifoulki cave (yellow circle^8^).

Speleothems can be precisely dated and provide high-resolution climate records of effective rainfall (i.e. the amount of precipitation that actually infiltrates into the soil) during the past^9^. Hence, stalagmites have been sampled from caves in Northern Morocco (Fig. 1), which is a key NAO region at the north-eastern side of the Azores Subtropical High. In this study, we present two new precisely dated high-resolution speleothem δ^18^O records (Cha2 and GP5) from Chaara and Piste caves respectively (Suppl. Text 1; Fig. S1). The purpose of this work is to provide new insights on decadal to centennial western Mediterranean hydro-climate variability and its relation with solar forcing during the last millennium.

## Results and Discussion

### GP5 and Cha2 speleothem records

The age model of the GP5 record is constructed using 23 Th/U dates, and the δ^18^O record covers the period ▯910–1960 AD with a short hiatus centered at approximately 960 AD. For the Cha2 record, the age model is based on 17 Th/U dates revealing that the sample spans the period 912–2000 AD with a hiatus from 1555 to 1736 AD (Suppl. Text 2; Fig. S2). The average age uncertainties are 8.6 and 6.5 years for GP5 and Cha2, respectively (Table S1). The growth rate of GP5 is higher than for Cha2 with 0.31 vs. 0.09 mm/yr, respectively. The δ^18^O sampling resolution is also relatively high in both records, with a mean resolution of 5 and 7.5 years for GP5 and Cha2, respectively. However, during a period of slow growth in the Cha2 record (1250–1460 AD), the δ^18^O sampling resolution drops off to 25 years. Therefore, we refrain from the interpretation of periodicities lower than 25 years in the statistical analyzes below.

**Figure 2:**
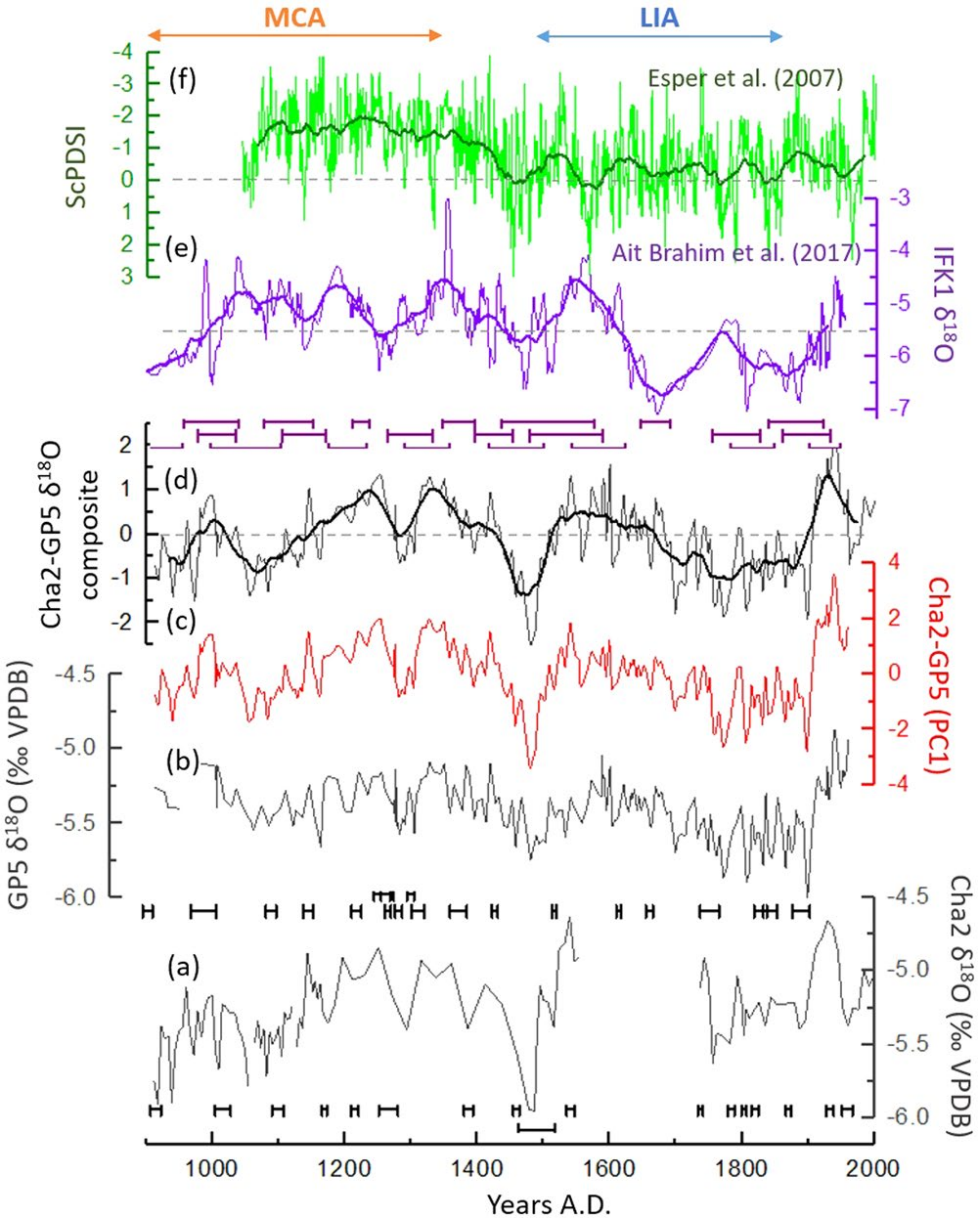
Comparison of the Cha2 (**a**) and GP5 (**b**) δ^18^O records with other paleo-records; (**c**) PC1 of Cha2 and GP5 δ^18^O records; (**d**) composite record of Cha2 and GP5 δ^18^O records; (**e**) IFK1 stalagmite δ^18^O record^8^; (**f**)tree-rings ScPDSI record^12,13^. Bold curves correspond to the 50-year moving average of each time-series.

The average δ^18^O values of GP5 and Cha2 records are −5.39‰ and −5.27‰ respectively. The δ^18^O records of both GP5 and Cha2 show similar trends and replicate very well (Fig. 2a,b). However, GP5 has a smaller δ^18^O amplitude compared to Cha2, which may be explained by longer water residence time and coincident mixing of several years of precipitation resulting in a smoothing effect^10^. The principal component analysis (PCA) between the GP5 and Cha2 δ^18^O time-series was performed to extract the common variability between both records. A composite record of GP5 and Cha2 δ^18^O records was also calculated (see Methods). The first component of the PCA (PC1) represents 67% of the common variability of both records. Furthermore, PC1 is almost identical to the composite of GP5 and Cha2 (Fig. 2c,d).

Based on previous monitoring work carried out in the caves region^11^, modern precipitation δ^18^O values are well correlated to rainfall amount (r ≥ 0.7) on seasonal timescales, whereas winter rainfall amount is correlated to the NAO index (r = 0.66). The GP5 and Cha2 δ^18^O records are thus considered proxies for effective rainfall. Therefore, we use the composite record in the discussion below as a representative record of effective rainfall variability during the last millennium in Northern Morocco, whereas negative values reflect humid conditions and vice-versa.

The composite δ^18^O record shows substantial alternations of dry and humid conditions on decadal to centennial time-scales (Fig. 2d). The Medieval climate anomaly (MCA) and the Little Ice Age (LIA) are defined as ∼900–1350 AD and ∼1500–1850 AD, respectively, based on previous paleoclimate studies in Morocco^8,12,13^. The general trend of our δ^18^O composite record supports a previously published speleothem δ^18^O record from Ifoulki cave from southwestern Morocco^8^ (Fig. 2e). Taking into account the age uncertainties in both records and their different resolutions, more negative δ^18^O values prevailed during the first part of the MCA (~910–990 AD), the period from ~1270 AD to ~1310 AD with a peak centered around 1290 AD, the period from ~1430 AD to ~1510 AD with a peak centered around 1480 AD, and from the second part of the LIA to the early 20th century (~1680–1910 AD). On the opposite, more positive δ^18^O values prevailed in both records during the second part of the MCA (~1140–1260 AD and ~1310–1380 AD), the first part of the LIA (~1520–1680 AD) and during the 20th century with a positive peak centered around 1940 AD. However, the two records show an anti-phase around 1050 and 1780 AD.

The Cha2-GP5 δ^18^O composite record also shows some coherent variations with a tree-ring Self-calibrating Palmer Drought Severity Index (ScPDSI) record from Northern Morocco (Fig. 2f)^12,13^, especially after the MCA. A pronounced shift towards wetter conditions after 1400 AD is in phase with the onset towards more humid conditions recorded in the Cha2-GP5 record. During the MCA/LIA transition and the LIA, peaks of wet conditions (around 1480 AD, 1570 AD, 1700 AD, 1770 AD and 1850 AD) seem to be synchronous with negative δ^18^O values in the composite record on decadal time-scale. However, during the MCA, the ScPDSI shows overall drier conditions compared to the Cha2-GP5 record. These differences might be related to the different seasons recorded in Cha2-GP5 speleothems (winter) and the tree-rings ScPDSI (February–June).

### North Atlantic climate variability

Previous studies based on instrumental and climate model data have shown that the Western Mediterranean climate is modulated by rainfall variability linked to the NAO^1,2^. Intervals with increased rainfall in Morocco are indeed related to negative phases of the NAO at inter-annual timescale. At decadal timescale, both AMO and NAO low frequency modulation exert a large influence on hydro-climate variations in the North Atlantic sector.

**Figure 3:**
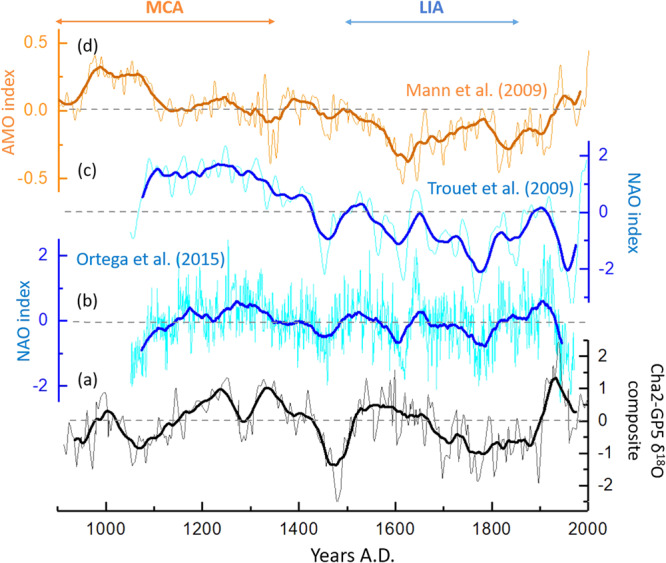
Comparison of the composite record of Cha2 and GP5 δ^18^O records (**a**) with the reconstructed NAO index^6^ (**b**), the reconstructed NAO index^14^ (**c**) and the reconstructed AMO index^18^ (**d**). Bold curves correspond to the 50-year moving average of each time-series.

Comparison of our record with instrumental NAO index during the common period of both records (1850–2000 AD) shows a high consistency (r = 0.63; Fig. S3), taking into account the age uncertainties in our speleothems records. Specifically, phases with a negative NAO index coincide with lower speleothem δ^18^O values, and hence wet conditions. For the last millennium, to a good extent, the lower δ^18^O values in our record (Fig. 3a) are synchronous with negative phases of the NAO (Fig. 3b,c) as shown by comparison with the NAO index reconstructions^6,14^. This is especially relevant for the negative peaks centered around 1050 AD, 1480 AD, 1610 AD and 1770 AD. However, several inconsistencies on longer time-scales can also be observed suggesting that another mechanism is involved as well in the region (e.g. AMO).

Positive AMO conditions were found to favor extensive cyclonic pressure anomalies over the Western Mediterranean, especially during the winter season^15^. Moreover, McCarthy *et al*.^16^ showed that the NAO influences the AMO through changes of the ocean circulation in the North Atlantic inter-gyre region. A positive AMO phase tends to result in more frequent negative NAO with a large influence on precipitation^15,17^. This would result in more moisture in-flow and wetter conditions in the Western Mediterranean during the winter season. However, comparison with the reconstructed AMO index^18^ (Fig. 3d) shows that the anti-phase relationship between NAO and AMO seems to be more complicated during the last millennium. Nevertheless, Redfit spectral analysis of our record (Fig. S4a) reveals significant multi-decadal periodicities similar to AMO. Other NAO reconstructions have shown similar periodicities to AMO for the last millennium as well^14,19^. Our findings are thus in line with former studies and brings further evidence that the multi-decadal variability of the NAO is the result of a link to AMO^19,20^. The multi-decadal imprint of AMO over the last millennium has recently been observed in a speleothem δ^18^O record from a cave in south-western Morocco^8^. The centennial-scale variability shown by the spectral analysis in the North Atlantic region has so far only been signaled in a few studies. A NAO-type 170-year quasi-periodicity was observed in a laminated sediment record from Greenland^19^. This variability has been suggested to be the result of centennial-scale solar forcing^21^. Recently, a multi-proxy lake record from the Middle Atlas in Morocco, also revealed that a multi-centennial-scale NAO-type pattern, related to solar variability, modulates Western Mediterranean climate^22^.

### Linkage to solar forcing

Redfit spectral analysis of our δ^18^O composite record shows strong centennial-scale periodicities at 199–174 years and 107–100 years, significant at the 95% confidence level. The 199–174 year periodicities in the spectral analysis are close to the Vries-Suess solar cycle^23^. Similar high power periodicities around 200 years were also reported in a speleothem record in Southwestern Morocco^8^. These results suggest that δ^18^O variability responds to solar forcing through NAO as suggested above. High power 200-year periodicities were also recorded in the CaCO_3_ record from Lake Sidi Ali in northern Morocco^22^. Periodicities similar to the 100-70 year Gleissberg cycle^24^ can also be identified in our record and in the ScPDSI record (Fig. S4b). However, the ScPDSI record does not show significant periodicities over 200 years. This might imply that the 200-year cycle is mainly recorded in the winter season, rather than the February-June season.

The NAO-response to solar activity has been observed and modeled in a number of studies^25–29^. In particular, the atmosphere–ocean response^30,31^ and the weakening of westerly winds during winter at times of solar minima are associated with patterns similar to a negative phase of the NAO^32,33^. The mechanism between the NAO and solar forcing has been proposed by a number of studies, which describe that at times of reduced solar irradiance, the downward-propagating effects triggered by changes in stratospheric ozone induce a cooling of the high northern latitude atmosphere and a southward shift of the Northern Subtropical Jet^29,30,34,35^.

**Figure 4:**
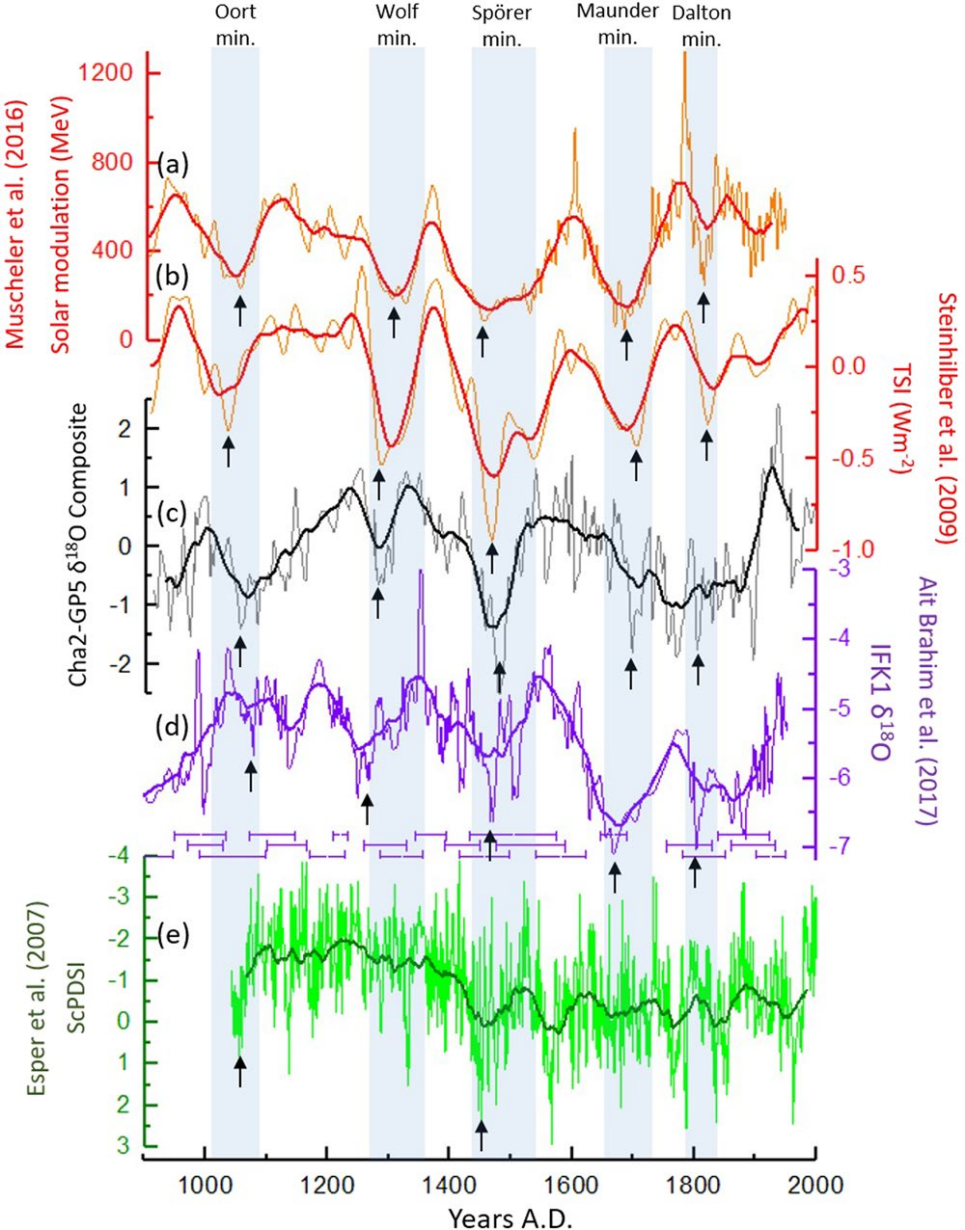
Comparison of the solar modulation reconstruction^37^ (**a**) and the total solar irradiance (TSI) reconstruction^36^ (**b**) with the Cha2-GP5 δ^18^O composite record (**c**), the Ifoulki cave (IFK1) stalagmite δ^18^O record^8^ (**d**) and the tree-rings ScPDSI record^12,13^ (**e**). Blue shading indicates periods of low solar activity: Oort minimum (1010–1090 AD), Wolf minimum (1280–1350 AD), Spörer minimum (1450–1550 AD), Maunder minimum (1645–1715 AD) and the Dalton minimum (1790–1820 AD). Black arrows indicate peaks of low solar activity and their corresponding peaks of wet conditions in Morocco.

Wavelet analysis of the composite record further indicates that the robust 200-year cycle is pervasive throughout the entire record (Fig. S5). Importantly, a clear in-phase relationship is observed between our composite δ^18^O record and the widely used total solar irradiance (TSI) reconstruction^36^ and a recently revised solar modulation record^37^. Especially, the Spörer minimum coincides with the lowest δ^18^O value in our record. The negative peaks during the Oort, Wolff, Maunder and Dalton solar minima also seem to coincide with more negative speleothem δ^18^O values in Morocco. However, the Maunder and Dalton minima are less pronounced in the composite record (Fig. 4a–c). When taking age uncertainties into account, the Ifoulki cave δ^18^O record from Morocco^8^ also shows low δ^18^O value during grand solar minima, with exception to the Oort minimum which is less noticeable (Fig. 4d). The tree-rings ScPDSI (Fig. 4e) also reveals wet conditions during the grand solar minima. However, given the robust age model of the ScPDSI record, only the low solar activity peaks during Oort and Spörer minima seem to be synchronized with wet conditions. This reinforces our assumption that the centennial solar forcing is mainly captured during the winter season.

**Figure 5:**
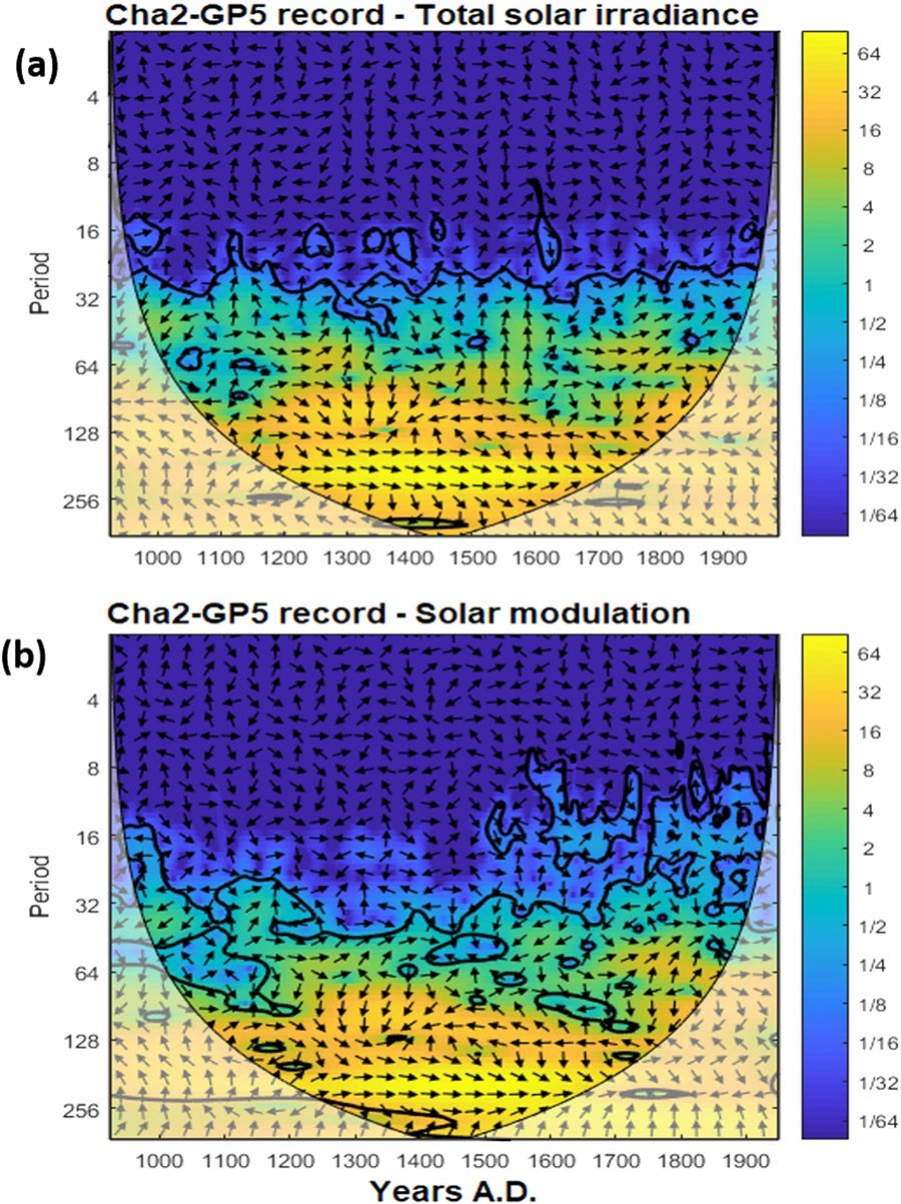
Cross-wavelet analyses of Cha2-GP5 δ^18^O record with the Total solar irradiance^36^ (**a**) and with the solar modulation reconstructions^37^ (**b**). Black arrows indicate phasing of the two records (left: anti-phase, right: in-phase, down: X leading Y by 90 degrees, Y leading X by 90 degrees).

In addition, the cross-wavelet analyzes of our record and both solar reconstructions shows a clear correlation with the highest power at periodicities of approximately 200 years (Fig. 5a,b). Strong power is also observed on the multi-decadal timescale, yet the phasing oscillates between both records, which might be suggestive of a less consistent influence of the solar signal on multi-decadal time scales. We therefore focus on the 200-year time scale variability, which we demonstrated has the most coherent and persistent response to solar forcing. This is consistent with paleoclimate data^38^ which suggest a shift toward low NAO state during periods of reduced solar forcing. Moreover, during the Maunder Minimum for example, model results and reconstructions suggest that solar-forced regional climate changes appeared predominantly as a shift towards a more negative NAO index^38,39^. Such negative phases of NAO-like conditions are associated with a southward shift of the northern subtropical jet, which drives the moisture flow towards the Western Mediterranean.

## Conclusions

A speleothem δ^18^O composite record of the last millennium from two high-resolution Th/U dated stalagmites from two caves in Morocco is presented and discussed. The link between NAO/AMO activity and hydro-climate variability described by our record is confirmed by previous monitoring work and historical data. Hence, the δ^18^O variability is interpreted as a result of the combination of NAO and AMO.

Our δ^18^O record reveals centennial cycles similar to well-known solar cycles. Interestingly, a 200-year cycle persists throughout the entire record. Comparison with solar forcing reconstructions shows a striking consistency, and the cross-wavelet further shows a clear correlation with the highest power at periodicities similar to the Vries-Suess solar cycle. Low irradiance periods during well know solar minima periods coincide with negative δ^18^O peaks, consistent with model results that suggest that the NAO responds to solar activity.

Hence, we have provided new proxy evidence for centennial-scale solar forcing. We also highlight the potential of speleothems and the relevance of using high resolution proxy records with robust age models to investigate potential teleconnections and coupling with forcing mechanisms to confirm climate models.

## Methods

Two new high resolution δ^18^O records from two aragonite stalagmites are presented. The two stalagmites were collected in 2010 from two adjacent caves that are only a few 100 m apart (33°57′21″N, 4°14′46″ W, 1260 m.a.s.l.). Stalagmite Cha2 was collected below an active drip site at 300 m distance of the entrance of Chaara cave and is 60 cm long. Stalagmite GP5 was collected below an active drip site at 50 m distance of the entrance of Grotte de Piste^13^ and is 78 cm long (Fig. S6).

The present day climate of the cave sites is characterized by average annual rainfall around 1267 mm at Bab Bou Idir station with 85% of rainfall amount falling during the winter season (Suppl. Text 1).

The stalagmite chronologies are based on 17 Th/U dates for Cha2 and 23 Th/U dates and the detection of the ^14^C bomb peak for GP5 (Suppl. Text 2). Age depth modeling was performed using the StalAge algorithm^40^. Based on the detection of the ^14^C bomb peak in stalagmite GP5, the year 1964 was assigned to 0.2 mm distance from top^13^.

Our paleoclimate records are based on 123 δ^18^O samples from Cha2 and 204 samples from GP5 using conventional Isotope Ratio Mass Spectrometers (Suppl. Text 3). The oxygen isotope values were corrected using a calcite acid fractionation factor, but were converted to aragonite oxygen isotope values after evaluation with the factor^41^.

In order to extract the common variability between Cha2 and GP5 records, we performed a principal component analysis (PCA). For this purpose, time-series were interpolated to create annual resolution and then normalized. A composite record was also constructed by averaging the normalized δ^18^O values obtained from the time series of Cha2 and GP5. In order to detect the most significant periodicities documented in time-series, REDFIT^42^ and spectral analysis were carried out using the PAST software^43^. Wavelet and cross-wavelet analyses^44^ were also performed to check significant periodicities and high common power between our records and other paleoclimate reconstructions.

## Electronic supplementary material

Supplementary material

## Data Availability

The data presented in this research article can be downloaded from the NOAA paleoclimate database https://www.ncdc.noaa.gov/paleo-search/study/25970.
