# Publisher Correction: Multi-decadal to centennial hydro-climate variability and linkage to solar forcing in the Western Mediterranean during the last 1000 years

**DOI:** 10.1038/s41598-020-69147-z

**Published:** 2020-07-16

**Authors:** Yassine Ait Brahim, Jasper A. Wassenburg, Francisco W. Cruz, Abdelfettah Sifeddine, Denis Scholz, Lhoussaine Bouchaou, Emilie P. Dassié, Klaus P. Jochum, R. Lawrence Edwards, Hai Cheng

**Affiliations:** 1 Insistute for Global Environmental Change, Xi’an Jiaotong University, Xi’an, China; 2 Climate Geochemistry Department, Max Planck Institute for Chemistry, Mainz, Germany; 3 Instituto de de Geociências, Universidade de São Paulo, São Paulo, Brazil; 4 IRD-Sorbonne Universités (UPMC, CNRS, MNHN) UMR LOCEAN, Centre IRD, Bondy, France; 5 Institute of Geoscience, University of Mainz, Mainz, Germany; 6 Laboratory of Applied Geology and Geo-Environment, Ibn Zohr University, Agadir, Morocco; 7 EPOC, UMR 5805, CNRS, University of Bordeaux, Pessac, France; 8 Department of Earth Sciences, University of Minnesota, Minneapolis, MN 55455 USA

Correction to: *Scientific Reports* 10.1038/s41598-018-35498-x, published online 28 November 2018.

The original version of this Article contained errors.

The author R. Lawrence Edwards was omitted from the Supplementary Material author list and the Author Contributions Statement. The Author Contributions statement now reads:

“Y.A.B. and J.A.W. prepared the manuscript. Y.A.B. and J.A.W. carried out δ18O analyses, Th/U dating and XRD analyses. F.W.C., H.C., D.S., R.L.E., and K.P.J. provided lab support. A.S., L.B., J.A.W., F.W.C and H.C. carried out the fieldwork. H.C. directed the project. E.P.D. and all authors provided comments on the manuscript and assisted with discussions.”

Additionally, the following affiliation was omitted from the Supplementary Information file:

“Department of Earth Sciences, University of Minnesota, Minneapolis, MN 55455, USA.”

Furthermore, the Data Availability Statement has been updated from:

"After acceptance of the manuscript, the data produced here will be published in a public database such as NOAA or PANGAEA."

to:

“The data presented in this research article can be downloaded from the NOAA paleoclimate database https://www.ncdc.noaa.gov/paleo-search/study/25970)”.

In addition, Yassine Ait Brahim was omitted as being a corresponding author. Correspondence and request for materials can also be addressed to aitbrahim@xjtu.edu.cn.

Lastly, in the original HTML version of this Article, R. Lawrence Edwards was incorrectly affiliated with ‘Climate Geochemistry Department, Max Planck Institute for Chemistry, Mainz, Germany’. The correct affiliation is listed below:

“Department of Earth Sciences, University of Minnesota, Minneapolis, MN 55455, USA.”

These errors have been corrected in the HTML and PDF versions of the Article, as well as in the Supplementary Material.

