# Supplementary material for: Multi-decadal to centennial hydro-climate variability and linkage to solar forcing in the Western Mediterranean during the last 1000 years
Source: Sci Rep. 2018 Nov 28;8:17446. doi: 10.1038/s41598-018-35498-x (PMC6261964; doi:10.1038/s41598-018-35498-x)
Supplement: Supplementary file 1 — Supplementary material [file 41598_2018_35498_MOESM1_ESM.docx]

**Supplementary material:**

**Multi-decadal to Centennial hydro-climate variability and linkage to solar forcing in the Western Mediterranean during the last 1000 years.**

Yassine Ait Brahim^1*^, Jasper A. Wassenburg^2^, Francisco W. Cruz^3^, Abdelfettah Sifeddine^4^, Denis Scholz^5^, Lhoussaine Bouchaou^6^, Emilie P. Dassié^7^, Klaus P. Jochum^2^, R. Lawrence Edwards^8^, Hai Cheng^1,8*^

1. Insistute for Global Environmental Change, Xi’an Jiaotong University, Xi’an, China

2. Climate Geochemistry Department, Max Planck Institute for Chemistry, Mainz, Germany

3. Instituto de de Geociências, Universidade de São Paulo, São Paulo, Brazil

4. IRD-Sorbonne Universités (UPMC, CNRS, MNHN) UMR LOCEAN, Centre IRD, Bondy, France

5. Institute of Geoscience, University of Mainz, Mainz, Germany

6. Laboratory of Applied Geology and Geo-Environment, Ibn Zohr University, Agadir, Morocco

7. EPOC, UMR 5805, CNRS, University of Bordeaux, Pessac, France

8. Department of Earth Sciences, University of Minnesota, Minneapolis, MN 55455, USA

Correspondence and requests for materials should be addressed to Y.A. (email: [aitbrahim@xjtu.edu.cn](mailto:aitbrahim@xjtu.edu.cn)) and/or H.C. (email: [cheng021@xjtu.edu.cn](mailto:cheng021@xjtu.edu.cn))

- **Supplementary text 1: Cave sites**

Both Chaara and Piste caves are developed in the Liassic carbonate rocks and are part of a complex of cave systems in the Middle Atlas region^1,2,3^. The present-day climate of the cave sites is characterized by average annual rainfall around 1267 mm at Bab Bou Idir station (1570 m asl). 85% of this annual rainfall is distributed during the winter season. The average maximum temperatures of the warmest month vary between 34.5°C at Taza station and 28°C at station Tazekka (1380 m.asl.). The average minimum temperatures of the coldest month vary between -2.8 °C at the station of Bab Bou Idir and 5.5°C at the Taza station. The vegetation in this massif is composed of the Atlas Cedar *(Cedrus atlantica)* forests, oaks (*Quercus rotundifolia*) and thuya (*Tetraclinis articulate*), but the massif also presents more than a dozen of other tree isolated feet species.

- **Supplementary text 2: Age model**

Th/U analyses of 17 samples from Cha2 were carried out at the Minnesota Isotope Laboratory, using a Neptune multi-collector inductively coupled plasma mass spectrometer (MC-ICPMS)^4^. Chemical procedures used to separate uranium and thorium fractions for ^230^Th dating are similar to those described by Edwards et al. (1987)^5^. The 2σ error associated with this method inferior at 1 %. For GP5, the age model is based on 23 Th/U dates, and the detection of the ^14^C bomb peak in the top of GP5^6^. Eight Th/U dates were analyzed at the GEOMAR Helmholtz Center for Ocean Research Kiel, Germany^6^, and 15 new ages were analyzed with a Nu MC-ICP-MS at the Max Planck Institute for Chemistry (MPIC), Mainz, Germany. Chemical separation procedures are described in Yang et al. (2015)^7^, analytical setup and methods are provided by Obert et al. (2016)^8^.

For two samples of stalagmite GP5, an inter laboratory comparison between Kiel, Mainz and the Xi’an Jiaotong Isotope Laboratory was performed. Three aliquots of the same sample powder was analyzed in all three laboratories. All ages are in agreement within error (Fig. S7). As described by Wassenburg et al. (2013)^6^, the corresponding error bars include the uncertainty of the thickness of the sample hole (i.e., 2.5-4 mm). For the new ages determined in the framework of this study, the whole sample powder was used for the analyses. Therefore, the assigned distance from top exactly corresponds to the middle of the sample hole, and the uncertainty was not increased. The age-depth model was constructed for each speleothem using the StalAge algorithm^9^.

- **Supplementary text 3: Oxygen isotope analysis**

In order to produce a high resolution δ^18^O record that spans the last 1000 years, a total of 123 samples were collected along the top 61 mm of the growth axis of the Cha2 stalagmite. Sampling was done at a resolution of 0.5 mm using a Sherline micro-drill model 4000, coupled to an automated 3D stage. Speleothem powder samples were analyzed by an Isotope Ratio Mass Spectrometer (IRMS) in the laboratory of karst systems at the University of Sao Paulo. The analytical error is 0.1‰.

204 samples were hand drilled with a 0.5 mm dentist drill from the first 185 mm of the growth axis of stalagmite GP5, which corresponds to an average resolution of approx. 0.9 mm. These samples were analyzed at the Ruhr University Bochum, with a Finnigan MAT 253 isotope ratio mass spectrometer coupled to a Gasbench. Average precision is 0.14‰ for δ^18^O. Results are reported relative to the Vienna Pee Dee belemnite (VPDB) standard.

*
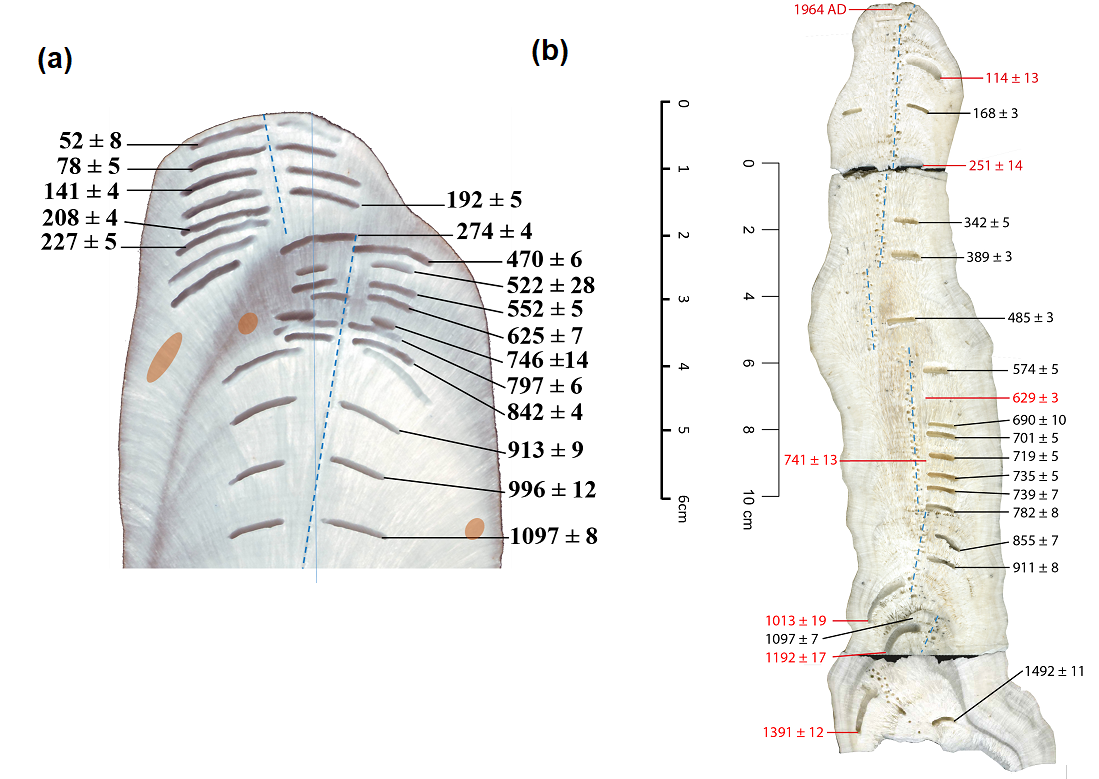
*

*Fig. S1: Cha2 (a) and GP5 (b) speleothems indicating the location of Th/U dating samples and sample transects for oxygen isotopes (dashed blue). Orange shading indicates the approximate sample positions for the X-Ray Diffraction. Red ages in (b) were published in Wassenburg et al. (2013)^6^.*

*
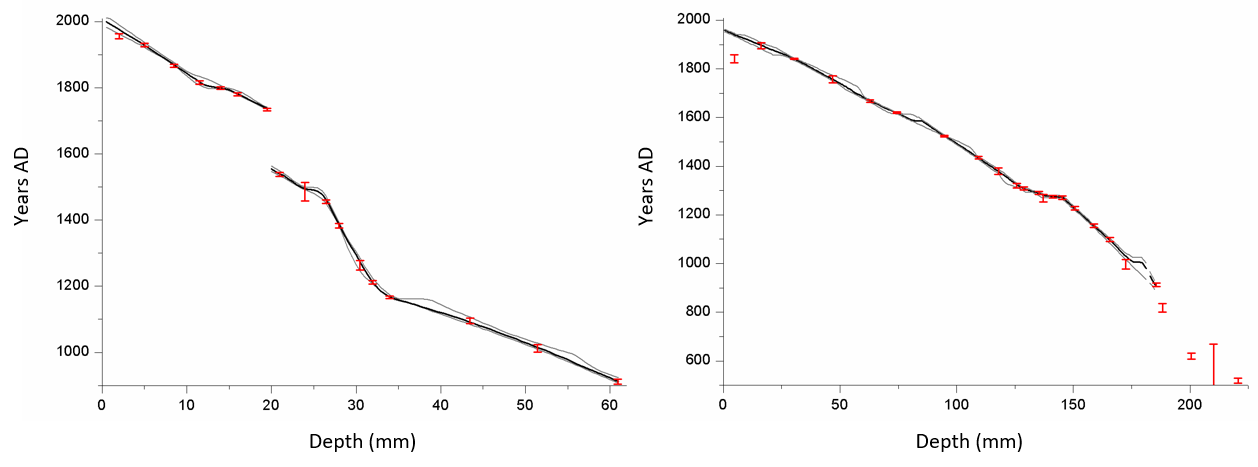
*

*Fig. S2: Chronological age models developed for Cha2 (a) and GP5 (b) based on StalAge Algorithm^9^. The gray lines represent the age model’s 95% confidence intervals.*


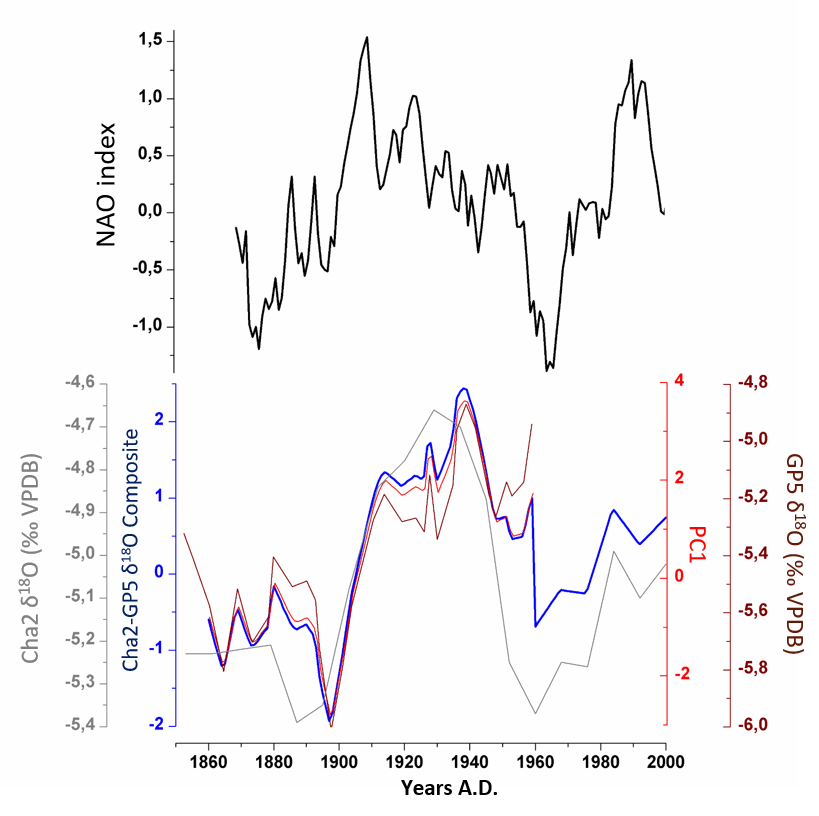


*Fig. S3: Comparison of the Instrumental NAO with Cha2 and GP5 speleothem records and their first axis of PCA and composite record for the common period (1850-2000 AD). The NAO index used here is station-based on the difference of normalized sea level pressure (SLP) between Lisbon, Portugal and Stykkisholmur/Reykjavik, Iceland.*

*
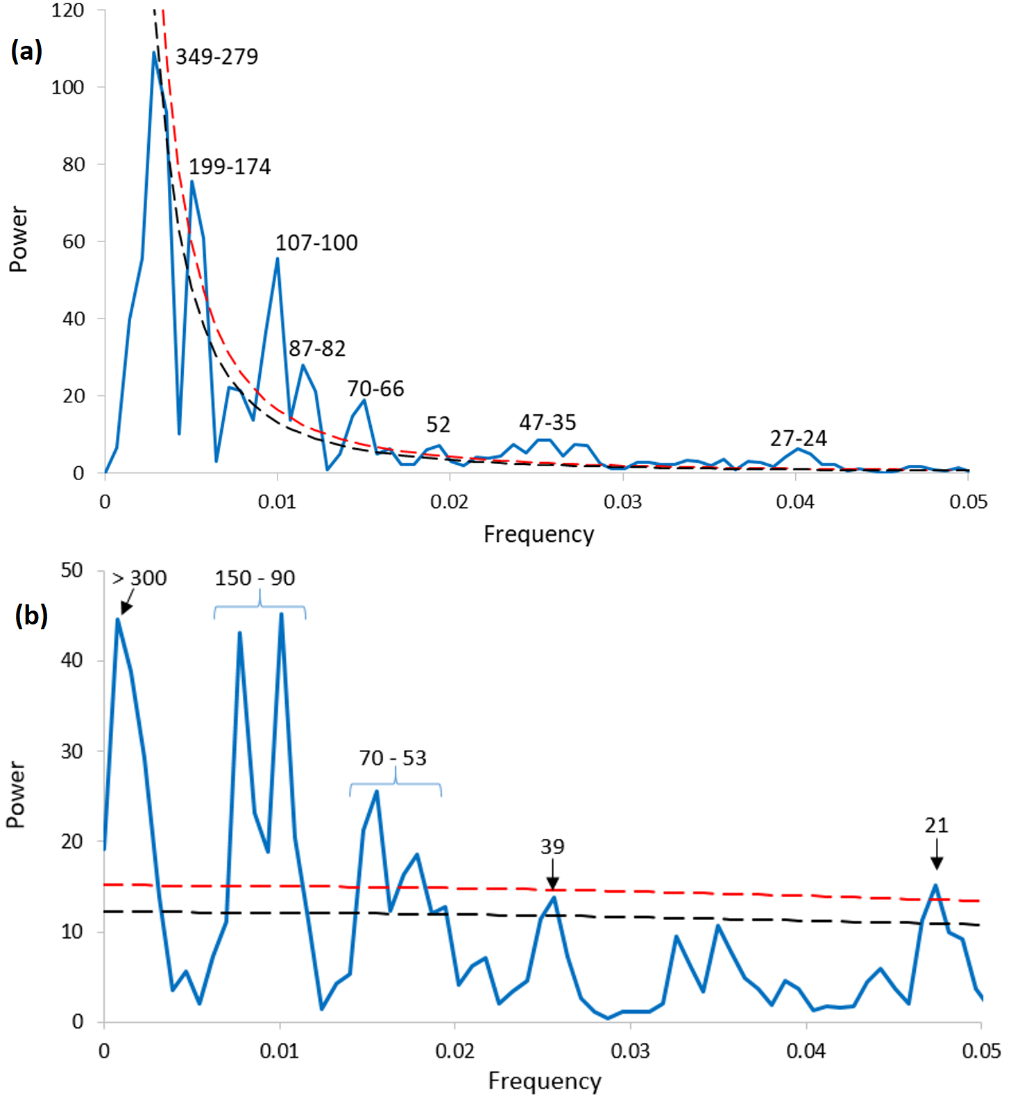
*

*Fig. S4: Redfit spectral analyses of Cha2-GP5 composite record (a) and the tree-rings ScPDSI record^10^. Dashed curves indicates the 95% (red curve), and the 90% (black curve) significance thresholds. The analysis was carried out using the PAST software^11^.*


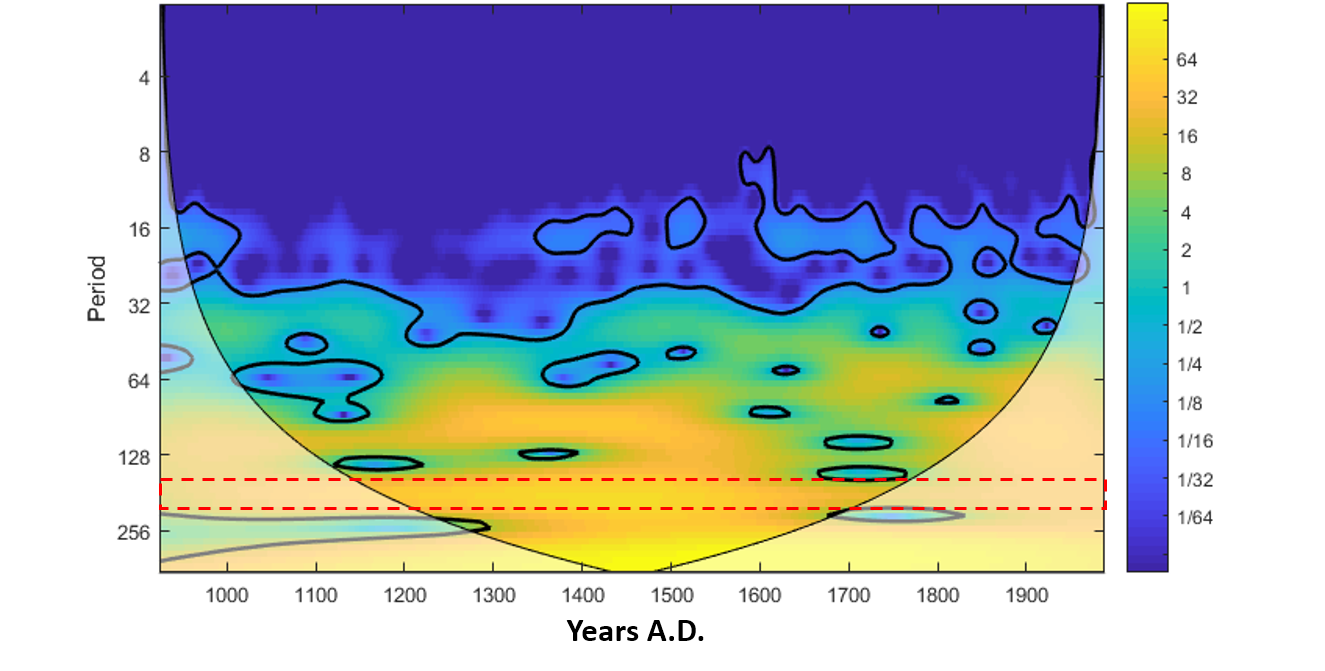


*Fig. S5: Continuous wavelet analysis of the Cha2-GP5 composite record. Dashed red rectangle indicates persistent high power periodicity around 200 years.*

*
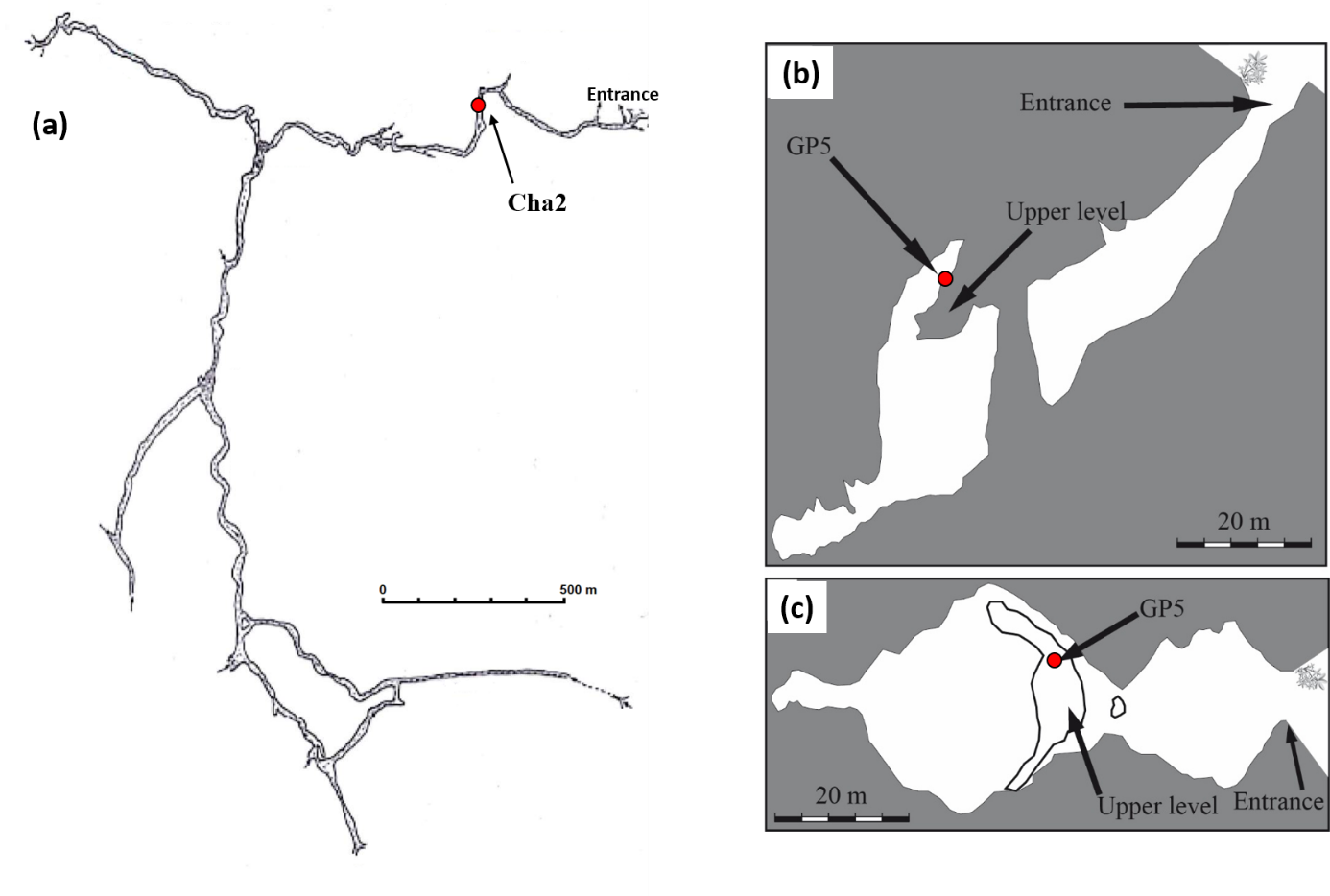
*

*Fig S6: cave maps of Chaara (a) (modified after Speleological Cave inventory of the Morocco, Rabat, unpublished, 1981) and Piste cave (modified after Wassenburg et al. 2013^6^). Red circles indicate the approximate location where Cha2 and GP5 stalagmites were collected in 2010.*

*
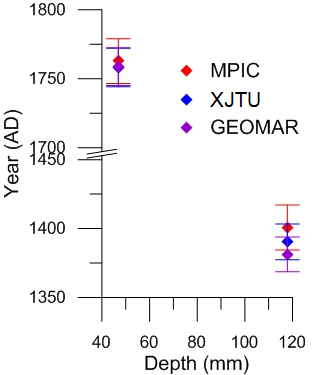
*

*Fig. S7: Inter- lab comparison of two Th/U dating samples between the GEOMAR Helmholtz Center for Ocean Research, Xi’an Jiaotong University (XJTU) Isotope Laboratory and the Max Planck Institute for Chemistry (MPIC).*

*Table S1: Cha2 and GP5 speleothems Th/U dating results. *δ^234^U = ([^234^U/^238^U]_activity_ – 1)x1000. ** δ^234^U_initial_ was calculated based on ^230^Th age (T), i.e., δ^234^U_initial_ = δ^234^U_measured_ x e^λ234xT^. Corrected ^230^Th ages assume the initial ^230^Th/^232^Th atomic ratio of 4.4 ±2.2 x10^-6^. Those are the values for a material at secular equilibrium, with the bulk earth ^232^Th/^238^U value of 3.8. Ages underlined were published in Wassenburg et al. (2013)^6^.*

|  | **Depth**  **(mm)** | **^238^U** | | **^232^Th** | | **^230^Th / ^232^Th** | | **δ^234^U*** | | **^230^Th / ^238^U** | | **^230^Th Age (yr)** | | **Year AD** | |
| --- | --- | --- | --- | --- | --- | --- | --- | --- | --- | --- | --- | --- | --- | --- | --- |
|  |  | **(ppb)** | | **(ppt)** | | **(atomic x10^-6^)** | | **(measured)** | | **(activity)** | | **(uncorrected)** | |  |  |
| **CHA2-1** | 2 | 481,5 | ±0,6 | 278 | ±6 | 77 | ±10 | 4355,0 | ±4,1 | 0,0027 | ±0,0004 | 55 | ±7 | **1964** | **±8** |
| **CHA2-2** | 5 | 356,3 | ±0,4 | 258 | ±5 | 92 | ±6 | 4383,6 | ±4,2 | 0,0040 | ±0,0002 | 82 | ±5 | **1938** | **±5** |
| **CHA2-3** | 8,5 | 524,6 | ±0,7 | 205 | ±4 | 297 | ±10 | 4381,1 | ±4,8 | 0,0071 | ±0,0002 | 143 | ±4 | **1875** | **±4** |
| **CHA2-12** | 11,5 | 1041 | ±2,3 | 721 | ±15 | 228 | ±7 | 4354,7 | ±7,4 | 0,0096 | ±0,0002 | 195 | ±5 | **1820** | **±5** |
| **CHA2-5** | 14 | 491,9 | ±0,6 | 209 | ±4 | 400 | ±11 | 4355,8 | ±4,3 | 0,0103 | ±0,0002 | 210 | ±3 | **1808** | **±4** |
| **CHA2-6** | 16 | 420,7 | ±0,5 | 178 | ±4 | 437 | ±13 | 4344,9 | ±4,3 | 0,0112 | ±0,0002 | 230 | ±5 | **1789** | **±5** |
| **CHA2-8** | 19,5 | 580,4 | ±0,7 | 192 | ±4 | 673 | ±17 | 4342,9 | ±4,5 | 0,0135 | ±0,0002 | 275 | ±4 | **1742** | **±4** |
| **CHA2-9** | 21 | 405,7 | ±0,5 | 380 | ±8 | 408 | ±9 | 4318,8 | ±4,1 | 0,0231 | ±0,0002 | 475 | ±5 | **1546** | **±6** |
| **CHA2-23** | 24 | 556 | ±1 | 3852 | ±77 | 64,8 | ±1,6 | 4311,8 | ±5,9 | 0,0272 | ±0,0004 | 560 | ±8 | **1494** | **±28** |
| **CHA2-10** | 26,5 | 387,4 | ±0,5 | 181 | ±4 | 951 | ±21 | 4306,8 | ±4,0 | 0,0270 | ±0,0002 | 555 | ±4 | **1464** | **±5** |
| **CHA2-11** | 28 | 310,1 | ±0,3 | 168 | ±4 | 924 | ±22 | 4294,6 | ±3,9 | 0,0304 | ±0,0003 | 628 | ±6 | **1391** | **±7** |
| **CHA2-29** | 30,5 | 405 | ±1 | 357 | ±8 | 670,1 | ±19,7 | 4218,0 | ±6,6 | 0,0358 | ±0,0007 | 751 | ±14 | **1270** | **±14** |
| **CHA2-12** | 32 | 387,0 | ±0,5 | 226 | ±5 | 1072 | ±24 | 4191,3 | ±4,1 | 0,0380 | ±0,0002 | 800 | ±5 | **1219** | **±6** |
| **CHA2-13** | 34 | 581,4 | ±0,7 | 220 | ±5 | 1738 | ±36 | 4161,9 | ±3,8 | 0,0399 | ±0,0001 | 844 | ±3 | **1174** | **±4** |
| **CHA2-42** | 43,5 | 432,2 | ±0,8 | 354 | ±7 | 870 | ±20 | 4154,8 | ±6,8 | 0,0433 | ±0,0004 | 918 | ±8 | **1103** | **±9** |
| **CHA2-50** | 51,5 | 512,7 | ±0,9 | 278 | ±7 | 1434 | ±38 | 4161,6 | ±7,1 | 0,0471 | ±0,0005 | 999 | ±11 | **1120** | **±12** |
| **CHA2-59** | 61 | 515,6 | ±0,9 | 297 | ±6 | 1475 | ±33 | 4120,2 | ±6,5 | 0,0515 | ±0,0003 | 1101 | ±7 | **918.5** | **±8** |
|  |  |  |  |  |  |  |  |  |  |  |  |  |  |  |  |
|  |  |  |  |  |  |  |  |  |  |  |  |  |  |  |  |
| **GP5 U4** | 4.8 | 4531.7 | 3.4 | 1158.2 | 1.9 | 577 | 5 | 4697.4 | ±5.6 | 0.008767 | 6.9E-05 |  |  | **1843** | **±17** |
| **GP5 U3.3** | 16.2 | 2974.9 | 1.7 | 479.2 | 3.6 | 635 | 6 | 4814.0 | ±4.2 | 0.006083 | 3.04E-05 |  |  | **1896** | **±13** |
| **GP5-30M** | 30.37 | 3433.8 | 28.0 | 383.4 | 3.8 | 1379 | 19 | 4816.8 | ±36.8 | 0.0093 | 0.0001 | 110 | 2 | **1842** | **±3** |
| **GP5 U3.2** | 46.9 | 3744.1 | 1.7 | 91.1 | 10.2 | 9235 | 1038 | 4811.8 | ±3.6 | 0.013364 | 2.57E-05 |  |  | **1759** | **±14** |
| **GP5-65M** | 62.94 | 3610.9 | 33.5 | 227.4 | 2.5 | 4889 | 70 | 4865.3 | ±45.3 | 0.0186 | 0.0002 | 283 | 5 | **1668** | **±5** |
| **GP5-U3.2.1M** | 74.28 | 3908.4 | 20.0 | 435.4 | 4.4 | 3217 | 40 | 5035.9 | ±7.7 | 0.0217 | 0.0002 | 329 | 4 | **1622** | **±3** |
| **GP5-U3.2.2M** | 94.76 | 3594.6 | 18.8 | 84.0 | 0.8 | 18993 | 199 | 5007.7 | ±9.3 | 0.0269 | 0.0002 | 425 | 3 | **1525** | **±3** |
| **GP5-U3.2.3M** | 109.42 | 2576.5 | 13.5 | 219.4 | 2.3 | 6046 | 72 | 4898.2 | ±9.7 | 0.0312 | 0.0003 | 514 | 5 | **1436** | **±5** |
| **GP5-U3.1** | 117.9 | 2299.5 | 0.9 | 64.0 | 10.4 | 19494 | 3170 | 4611.3 | ±3.1 | 0.032245 | 6.08E-05 |  |  | **1381** | **±13** |
| **GP5-124M** | 125.7 | 3761.6 | 38.4 | 182.3 | 3.0 | 13019 | 216 | 5015.9 | ±53.0 | 0.0382 | 0.0004 | 630 | 10 | **1320** | **±10** |
| **GP5-U0M** | 128.68 | 3275.7 | 16.8 | 28.5 | 1.2 | 71613 | 3047 | 4854.7 | ±8.3 | 0.0377 | 0.0003 | 641 | 6 | **1309** | **±5** |
| **GP5-U1M** | 135.27 | 3312.5 | 17.3 | 87.0 | 1.5 | 22421 | 395 | 4400.8 | ±7.1 | 0.0357 | 0.0003 | 659 | 5 | **1291** | **±5** |
| **GP5-U3.0** | 137.1 | 3266.0 | 2.8 | 160.7 | 3.5 | 12254 | 269 | 4298.1 | ±5.7 | 0.035858 | 9.31E-05 |  |  | **1269** | **±13** |
| **GP5-U1.1M** | 141.26 | 3231.6 | 16.5 | 43.0 | 1.4 | 46512 | 1496 | 4544.6 | ±6.9 | 0.0374 | 0.0002 | 675 | 5 | **1275** | **±5** |
| **GP5-U2M** | 145.21 | 2276.0 | 11.8 | 52.7 | 1.1 | 26715 | 593 | 4522.3 | ±7.0 | 0.0375 | 0.0003 | 679 | 7 | **1271** | **±7** |
| **GP5-U2.1M** | 150.63 | 2516.1 | 13.1 | 113.8 | 2.3 | 14833 | 323 | 4659.9 | ±8.3 | 0.0406 | 0.0004 | 722 | 8 | **1228** | **±8** |
| **GP5-158M** | 158.77 | 3364.3 | 23.4 | 289.5 | 2.7 | 8771 | 81 | 4817.5 | ±27.6 | 0.0457 | 0.0003 | 796 | 7 | **1155** | **±7** |
| **GP5-U2.2M** | 165.63 | 2073.9 | 13.6 | 546.9 | 5.4 | 3095 | 32 | 4908.9 | ±24.5 | 0.0493 | 0.0004 | 851 | 8 | **1099** | **±8** |
| **GP5 U3** | 172.4 | 4003.4 | 1.6 | 133.2 | 5.2 | 27405 | 1072 | 4869.3 | ±3.3 | 0.054241 | 0.000317 |  |  | **997** | **±19** |
| **GP5 U2.5aM** | 185.48 | 3666.3 | 24.1 | 498.1 | 5.2 | 7325 | 72 | 4991.4 | ±22.2 | 0.0602 | 0.0003 | 1037 | 8 | **913** | **±7** |
| **GP5 U2.5** | 188.1 | 3820.4 | 3.0 | 811.8 | 4.2 | 5231 | 32 | 5077.0 | ±6.2 | 0.066098 | 0.000227 |  |  | **818** | **±17** |
| **GP5 U2.4** | 200.5 | 3155.2 | 1.2 | 220.0 | 8.5 | 18121 | 703 | 4925.8 | ±2.9 | 0.075135 | 0.000097 |  |  | **619** | **±12** |
| **Artificial age** | 210 |  |  |  |  |  |  |  |  |  |  |  |  | **569** | **±100** |
| **GP5 U2.3eM** | 220.52 | 2991.0 | 19.7 | 53.9 | 1.5 | 70730 | 1921 | 4654.2024 | ±23.4 | 0.0771 | 0.0005 | 1432 | 11 | **518** | **±11** |

**References**

1. Sabaoui, A., Obda, K., & Laaouane, M. Potentialités géologiques du développement local du Moyen Atlas septentrional: structures, paysages et histoire géologique. Geomaghreb. **5**, 9–39 (2009).
2. Taous, A. et al. Karst et resources en eau au Moyen Atlas nord-oriental. Geomaghreb. **5**, 41–59 (2009).
3. Wassenburg, J.A. et al. Climate and cave control on Pleistocene/Holocene calcite-to-aragonite transitions in speleothems from Morocco: elemental and isotopic evidence. Geochim. Cosmochim. Acta. **92**, 23–47 (2012).
4. Cheng, H. et al. Improvements in 230Th dating, 230Th and 234U half-life values, and U–Th isotopic measurements by multi-collector inductively coupled plasma mass spectrometry. Earth Planet. Sci. Lett. **371–372**, 82–91 (2013).
5. Edwards R. L., Chen J. H. & Wasserburg G. J. 238U-234U- 230Th-232Th systematics and the precise measurement of time over the past 500,000 y. Earth Planet. Sci. Lett. **81**, 1752192 (1987).
6. Wassenburg, J.A. et al. Moroccan speleothem and tree ring records suggest a variable positive state of the North Atlantic Oscillation during the Medieval Warm Period. Earth Planet. Sci. Lett. **375**, 291–302 (2013).
7. Yang, Q., Scholz, D. & Jochum, K.P. et al. Lead isotope variability in speleothems – A promising new proxy for hydrological change? First results from a stalagmite from western Germany. Chemical Geology. **396**, 143–151 (2015).
8. Obert, J.C. et al. 230Th/U dating of Last Interglacial brain corals from Bonaire (southern Caribbean) using bulk and theca wall material. Geochimica et Cosmochimica Acta. **178**, 20-40 (2016).
9. Scholz, D. & Hoffmann, D.L. StalAge – an algorithm designed for construction of speleothem age models. Quat. Geochronol. **6**, 369–382 (2011).
10. Esper, J., Frank, D., Buntgen, U., Verstege, A., & Luterbacher, J. Long-term drought severity variations in Morocco. Geophys. Res. Lett. **34**, 5 (2007).
11. Hammer, Ø., Harper, D.A.T. & Ryan, P.D., PAST: paleontological statistics soft-ware package for education and data analysis. Palaeontol. Electronica. 4, 9 (2001).
